# Supplementary material for: Environmentally triggered shifts in steelhead migration behavior and consequences for survival in the mid-Columbia River
Source: PLoS One. 2021 May 10;16(5):e0250831. doi: 10.1371/journal.pone.0250831 (PMC8109777; doi:10.1371/journal.pone.0250831)
Supplement: S1 Table — For fish tagged and released in the Clearwater River, the fish from the South Fork Clearwater and Middle Fork Clearwater, as well as those from Dworshak National Fish Hatchery and Lolo Creek, were considered B-index. For the Salmon River, fish from the Middle Fork Salmon and South Fork Salmon were considered B-index. Additionally, both A-index and B-index hatchery fish have been released at the Pahsimeroi River trap in recent years. Hatchery fish at this trap were identified as either A-index or B-index based off of their stated stock name in the PTAGIS database. All other fish from the Salmon and Clearwater rivers were included in the Salmon/Clearwater A-index migration group. (DOCX) [file pone.0250831.s001.docx]

**S1 Table: Sample sizes by steelhead population groups, specific populations, and return years.** For fish tagged and released in the Clearwater River, the fish from the South Fork Clearwater and Middle Fork Clearwater, as well as those from Dworshak National Fish Hatchery and Lolo Creek, were considered B-index. For the Salmon River, fish from the Middle Fork Salmon and South Fork Salmon were considered B-index. Additionally, both A-index and B-index hatchery fish have been released at the Pahsimeroi River trap in recent years. Hatchery fish at this trap were identified as either A-index or B-index based off of their stated stock name in the PTAGIS database. All other fish from the Salmon and Clearwater rivers were included in the Salmon/Clearwater A-index migration group.

|  | **2004** | **2005** | **2006** | **2007** | **2008** | **2009** | **2010** | **2011** | **2012** | **2013** | **2014** | **2015** | **2016** | **Grand Total** |
| --- | --- | --- | --- | --- | --- | --- | --- | --- | --- | --- | --- | --- | --- | --- |
| **Middle Columbia** | **29** | **59** | **55** | **51** | **100** | **350** | **198** | **210** | **154** | **114** | **185** | **202** | **99** | **1,806** |
| Lower Yakima | 22 | 15 | 12 | 18 | 14 | 21 | 14 | 26 | 11 | 29 | 32 | 20 | 13 | 247 |
| Naches | 0 | 0 | 0 | 0 | 1 | 0 | 0 | 1 | 1 | 0 | 4 | 14 | 7 | 28 |
| Upper Yakima | 0 | 0 | 0 | 0 | 1 | 12 | 9 | 11 | 6 | 15 | 41 | 57 | 17 | 169 |
| Walla Walla | 7 | 44 | 43 | 33 | 84 | 317 | 175 | 172 | 136 | 70 | 108 | 111 | 62 | 1,362 |
| **Snake Early A-index** | **155** | **198** | **169** | **700** | **666** | **1,902** | **1,099** | **1,048** | **571** | **822** | **1,074** | **1,009** | **476** | **9,889** |
| Imnaha | 87 | 69 | 50 | 68 | 157 | 885 | 565 | 535 | 229 | 428 | 536 | 568 | 212 | 4,389 |
| Lower Grand Ronde | 6 | 0 | 1 | 11 | 7 | 257 | 139 | 195 | 108 | 97 | 178 | 137 | 123 | 1,259 |
| Lower Snake Asotin | 0 | 0 | 1 | 12 | 23 | 30 | 27 | 41 | 45 | 77 | 105 | 56 | 27 | 444 |
| Lower Snake Tucannon | 41 | 94 | 103 | 587 | 449 | 682 | 312 | 213 | 140 | 161 | 202 | 192 | 85 | 3,261 |
| Upper Grand Ronde | 10 | 17 | 10 | 13 | 15 | 30 | 32 | 30 | 21 | 36 | 37 | 37 | 17 | 305 |
| Wallowa | 11 | 18 | 4 | 9 | 15 | 18 | 24 | 34 | 28 | 23 | 16 | 19 | 12 | 231 |
| **Upper Columbia** | **3,102** | **5,485** | **6,618** | **1,087** | **513** | **1,088** | **744** | **960** | **874** | **672** | **725** | **692** | **232** | **22,792** |
| Entiat | 0 | 0 | 3 | 8 | 7 | 75 | 73 | 55 | 24 | 42 | 65 | 55 | 34 | 441 |
| Methow | 740 | 1,862 | 3,080 | 464 | 15 | 118 | 64 | 317 | 303 | 321 | 283 | 287 | 102 | 7,956 |
| Okanogan | 115 | 350 | 463 | 73 | 17 | 9 | 9 | 117 | 136 | 92 | 148 | 134 | 62 | 1,725 |
| Wenatchee | 389 | 404 | 400 | 334 | 474 | 886 | 598 | 471 | 411 | 217 | 229 | 216 | 34 | 5,063 |
| Ringold Hatchery | 1,858 | 2,869 | 2,672 | 208 | 0 | 0 | 0 | 0 | 0 | 0 | 0 | 0 | 0 | 7,607 |
| **Sal/Clear A-index** | **27** | **21** | **20** | **48** | **59** | **681** | **457** | **549** | **280** | **212** | **369** | **223** | **138** | **3,084** |
| Chamberlain Creek | 13 | 5 | 2 | 5 | 9 | 9 | 0 | 0 | 0 | 0 | 0 | 0 | 0 | 43 |
| Lemhi | 0 | 1 | 0 | 2 | 4 | 41 | 25 | 47 | 25 | 28 | 25 | 29 | 13 | 240 |
| Little Salmon | 1 | 2 | 3 | 5 | 17 | 371 | 192 | 172 | 66 | 57 | 116 | 63 | 45 | 1,110 |
| Lower Clearwater | 2 | 6 | 11 | 25 | 13 | 36 | 41 | 58 | 54 | 42 | 71 | 79 | 31 | 469 |
| Pahsimeroi | 0 | 0 | 0 | 1 | 0 | 30 | 19 | 6 | 1 | 2 | 2 | 3 | 0 | 64 |
| Upper Salmon | 11 | 7 | 4 | 10 | 16 | 194 | 180 | 266 | 134 | 83 | 155 | 49 | 49 | 1,158 |
| **Sal/Clear B-index** | **59** | **80** | **72** | **83** | **261** | **287** | **1,009** | **965** | **706** | **428** | **752** | **516** | **706** | **5,924** |
| Lochsa | 20 | 27 | 17 | 22 | 60 | 86 | 154 | 80 | 49 | 47 | 207 | 86 | 42 | 897 |
| Lower Clearwater | 0 | 3 | 0 | 5 | 5 | 4 | 23 | 14 | 4 | 0 | 8 | 14 | 53 | 133 |
| Dworshak Hatchery | 9 | 7 | 10 | 7 | 23 | 47 | 314 | 275 | 265 | 70 | 179 | 124 | 240 | 1,570 |
| Lower Middle Fork Salmon | 0 | 3 | 7 | 4 | 8 | 46 | 21 | 23 | 20 | 40 | 30 | 17 | 0 | 219 |
| Pahsimeroi | 0 | 0 | 0 | 0 | 0 | 0 | 0 | 8 | 47 | 24 | 57 | 49 | 63 | 248 |
| Selway | 6 | 2 | 2 | 1 | 4 | 4 | 1 | 0 | 0 | 0 | 0 | 0 | 0 | 20 |
| South Fork Clearwater | 13 | 27 | 24 | 37 | 148 | 54 | 346 | 433 | 294 | 212 | 186 | 191 | 283 | 2,248 |
| South Fork Salmon | 6 | 6 | 7 | 4 | 10 | 21 | 39 | 30 | 15 | 30 | 75 | 25 | 13 | 281 |
| Upper Middle Fork Salmon | 2 | 0 | 4 | 0 | 1 | 0 | 1 | 4 | 4 | 0 | 6 | 2 | 0 | 24 |
| Upper Salmon | 3 | 5 | 1 | 3 | 2 | 25 | 110 | 98 | 8 | 5 | 4 | 8 | 12 | 284 |
| **Total** | **3,372** | **5,843** | **6,934** | **1,969** | **1,599** | **4,308** | **3,507** | **3,732** | **2,585** | **2,248** | **3,105** | **2,642** | **1,651** | **43,495** |
