# Supplementary material for: Environmentally triggered shifts in steelhead migration behavior and consequences for survival in the mid-Columbia River
Source: PLoS One. 2021 May 10;16(5):e0250831. doi: 10.1371/journal.pone.0250831 (PMC8109777; doi:10.1371/journal.pone.0250831)
Supplement: S2 Table — Shown are survival from Bonneville Dam-McNary Dam, mean travel time from Bonneville Dam-McNary Dam, median day of arrival at Bonneville Dam, proportion hatchery fish (vs. wild), mean ocean age, and proportion of fish that were transported. (DOCX) [file pone.0250831.s002.docx]

**S2 Table: Summary of run statistics by steelhead population groups and yea**r. Shown are survival from Bonneville Dam-McNary Dam, mean travel time from Bonneville Dam-McNary Dam, median day of arrival at Bonneville Dam, proportion hatchery fish (vs. wild), mean ocean age, and proportion of fish that were transported.

| Year | Middle Columbia | Snake Early A-run | Upper Columbia | Sal/Clear A-run | Sal/Clear B-run |  | Middle Columbia | Snake Early A-run | Upper Columbia | Sal/Clear A-run | Sal/Clear B-run |  | Middle Columbia | Snake Early A-run | Upper Columbia | Sal/Clear A-run | Sal/Clear B-run |
| --- | --- | --- | --- | --- | --- | --- | --- | --- | --- | --- | --- | --- | --- | --- | --- | --- | --- |
|  | ***Survival*** |  |  |  |  |  | ***Hatchery proportion of tagged fish*** | | |  |  |  | ***Median arrival day of year*** | | |  |  |
| 2004 | 0.79 | 0.85 | 0.73 | 0.74 | 0.92 |  | 0.00 | 0.35 | 1.00 | 0.41 | 0.39 |  | 197 | 213 | 226 | 224 | 252 |
| 2005 | 0.86 | 0.75 | 0.69 | 0.82 | 0.84 |  | 0.56 | 0.45 | 1.00 | 0.41 | 0.51 |  | 213 | 214 | 219 | 223 | 260 |
| 2006 | 0.80 | 0.79 | 0.73 | 0.80 | 0.86 |  | 0.58 | 0.67 | 1.00 | 0.30 | 0.49 |  | 214 | 219 | 224 | 227 | 250 |
| 2007 | 0.73 | 0.78 | 0.74 | 0.90 | 0.76 |  | 0.45 | 0.83 | 0.99 | 0.25 | 0.61 |  | 213 | 209 | 222 | 237 | 249 |
| 2008 | 0.78 | 0.77 | 0.76 | 0.90 | 0.75 |  | 0.76 | 0.70 | 0.95 | 0.36 | 0.68 |  | 205 | 206 | 201 | 220 | 251 |
| 2009 | 0.80 | 0.78 | 0.77 | 0.84 | 0.82 |  | 0.73 | 0.84 | 0.83 | 0.83 | 0.42 |  | 213 | 220 | 219 | 234 | 249 |
| 2010 | 0.86 | 0.77 | 0.79 | 0.86 | 0.77 |  | 0.41 | 0.75 | 0.77 | 0.82 | 0.78 |  | 196 | 207 | 204 | 231 | 252 |
| 2011 | 0.82 | 0.82 | 0.80 | 0.81 | 0.76 |  | 0.29 | 0.58 | 0.86 | 0.78 | 0.86 |  | 213 | 218 | 221 | 236 | 253 |
| 2012 | 0.86 | 0.78 | 0.80 | 0.80 | 0.78 |  | 0.32 | 0.48 | 0.91 | 0.70 | 0.87 |  | 215 | 217 | 216 | 231 | 257 |
| 2013 | 0.70 | 0.73 | 0.73 | 0.73 | 0.80 |  | 0.13 | 0.67 | 0.83 | 0.65 | 0.72 |  | 214 | 220 | 218 | 230 | 257 |
| 2014 | 0.81 | 0.76 | 0.77 | 0.77 | 0.82 |  | 0.18 | 0.67 | 0.77 | 0.72 | 0.57 |  | 216 | 218 | 218 | 237 | 257 |
| 2015 | 0.76 | 0.77 | 0.80 | 0.84 | 0.87 |  | 0.20 | 0.66 | 0.76 | 0.48 | 0.72 |  | 217 | 221 | 220 | 236 | 259 |
| 2016 | 0.78 | 0.71 | 0.74 | 0.84 | 0.81 |  | 0.43 | 0.72 | 0.69 | 0.64 | 0.91 |  | 208 | 215 | 216 | 236 | 262 |
| **Avg.** | **0.80** | **0.77** | **0.76** | **0.82** | **0.81** |  | **0.39** | **0.64** | **0.87** | **0.57** | **0.66** |  | **210** | **215** | **217** | **231** | **255** |
|  | ***Mean travel time*** | |  |  |  |  | ***Average age of tagged fish*** | | |  |  |  | ***Proportion of tags transported as juvenile*** | | | |  |
| 2004 | 57.57 | 37.48 | 20.84 | 30.20 | 15.91 |  | 1.24 | 1.47 | 1.00 | 1.59 | 1.93 |  | 0 | 0.10 | 0 | 0.52 | 0.19 |
| 2005 | 53.02 | 35.47 | 19.66 | 38.11 | 15.07 |  | 1.22 | 1.49 | 1.64 | 1.73 | 1.98 |  | 0 | 0.28 | 0 | 0.73 | 0.68 |
| 2006 | 53.50 | 38.07 | 18.58 | 68.88 | 30.34 |  | 1.38 | 1.31 | 1.35 | 1.25 | 1.81 |  | 0 | 0.19 | 0 | 0.60 | 0.49 |
| 2007 | 40.30 | 21.05 | 16.19 | 24.56 | 19.38 |  | 1.20 | 1.07 | 1.75 | 1.48 | 1.57 |  | 0 | 0.08 | 0 | 0.35 | 0.30 |
| 2008 | 68.21 | 28.97 | 14.38 | 36.13 | 17.10 |  | 1.23 | 1.26 | 1.63 | 1.20 | 1.92 |  | 0 | 0.18 | 0 | 0.22 | 0.18 |
| 2009 | 39.99 | 32.48 | 17.62 | 29.99 | 18.82 |  | 1.11 | 1.11 | 1.28 | 1.09 | 1.41 |  | 0 | 0.31 | 0 | 0.41 | 0.31 |
| 2010 | 49.03 | 30.32 | 16.02 | 27.13 | 21.73 |  | 1.68 | 1.43 | 1.80 | 1.54 | 1.98 |  | 0 | 0.30 | 0 | 0.36 | 0.36 |
| 2011 | 62.96 | 24.97 | 10.64 | 24.77 | 15.83 |  | 1.37 | 1.31 | 1.31 | 1.45 | 1.87 |  | 0 | 0.27 | 0 | 0.36 | 0.23 |
| 2012 | 55.34 | 28.48 | 11.60 | 28.19 | 17.61 |  | 1.74 | 1.62 | 1.68 | 1.59 | 1.98 |  | 0 | 0.28 | 0 | 0.42 | 0.27 |
| 2013 | 63.69 | 32.80 | 16.41 | 30.83 | 19.84 |  | 1.36 | 1.24 | 1.48 | 1.40 | 1.76 |  | 0 | 0.20 | 0 | 0.31 | 0.23 |
| 2014 | 56.49 | 36.08 | 15.14 | 25.99 | 16.29 |  | 1.55 | 1.50 | 1.72 | 1.47 | 1.93 |  | 0 | 0.29 | 0 | 0.35 | 0.16 |
| 2015 | 49.53 | 38.48 | 16.03 | 30.66 | 15.55 |  | 1.62 | 1.41 | 1.59 | 1.57 | 1.79 |  | 0 | 0.33 | 0 | 0.42 | 0.29 |
| 2016 | 55.11 | 35.84 | 15.30 | 24.62 | 13.47 |  | 1.87 | 1.83 | 2.01 | 1.99 | 2.02 |  | 0 | 0.30 | 0 | 0.43 | 0.27 |
| **Avg.** | **54.21** | **32.35** | **16.03** | **32.31** | **18.23** |  | **1.43** | **1.39** | **1.56** | **1.49** | **1.84** |  | **0** | **0.24** | **0** | **0.42** | **0.30** |
