# Supplementary material for: Environmentally triggered shifts in steelhead migration behavior and consequences for survival in the mid-Columbia River
Source: PLoS One. 2021 May 10;16(5):e0250831. doi: 10.1371/journal.pone.0250831 (PMC8109777; doi:10.1371/journal.pone.0250831)
Supplement: S3 Table — Variables considered included ocean age (A), reartype origin (O), juvenile transportation history (J), smoothers for river temperature s(T), river flow (F), dam spill (S), and arrival date at Bonneville Dam s(D), and a random effect for year y. Models including one or none of the smoothers s(D), s(F), s(S) were compared. Coefficient values given for intercept. Number of parameters represented by np. (DOCX) [file pone.0250831.s003.docx]

**S3 Table**: **AICc model selection table for the probability migration delay (*pDelay*)**. Variables considered included ocean age (*A*), reartype origin (*O*), juvenile transportation history (*J*), smoothers for river temperature s(*T*), river flow (*F*), dam spill (*S*), and arrival date at Bonneville Dam s(*D*), and a random effect for year *y*. Models including one or none of the smoothers s(*D*), s(*F*), s(*S*) were compared. Coefficient values given for intercept. Number of parameters represented by *np*.

| **Int.** | ***A*** | ***O*** | ***J*** | ***s(T)*** | ***s(F)*** | ***s(S)*** | ***s(D)*** | ***y*** | **np** | **delta AICc** |
| --- | --- | --- | --- | --- | --- | --- | --- | --- | --- | --- |
| *Middle Columbia* | | | | | | | | | | |
| 0.84 | + |  | NA | + |  |  | + | + | 17 | 0 |
| 0.72 | + | + | NA | + |  |  | + | + | 18 | 0.23 |
| 0.62 |  | + | NA | + |  |  | + | + | 17 | 1.17 |
| 0.74 |  |  | NA | + |  |  | + | + | 16 | 1.22 |
| *Snake Early A-index* | | | | | | | | | | |
| 0.08 | + | + | + | + |  |  | + | + | 21 | 0 |
| 0.11 |  | + | + | + |  |  | + | + | 20 | 0.93 |
| 0.11 | + |  | + | + |  |  | + | + | 20 | 1.48 |
| 0.16 |  |  | + | + |  |  | + | + | 19 | 3.96 |
| *Upper Columbia* | | | | | | | | | | |
| -0.91 | + |  | NA | + |  |  | + | + | 21 | 0 |
| -0.91 | + | + | NA | + |  |  | + | + | 22 | 1.54 |
| *Sal/Clear A-index* | | | | | | | | | | |
| 0.70 |  | + |  | + |  |  | + | + | 18 | 0 |
| 0.63 |  | + | + | + |  |  | + | + | 19 | 0.17 |
| 0.66 | + | + |  | + |  |  | + | + | 20 | 0.2 |
| 0.58 | + | + | + | + |  |  | + | + | 21 | 0.32 |
| *Sal/Clear B-index* | | | | | | | | | | |
| -0.85 | + |  | + | + |  |  | + | + | 19 | 0 |
| -0.85 | + | + | + | + |  |  | + | + | 20 | 1.95 |
