# Supplementary material for: Environmentally triggered shifts in steelhead migration behavior and consequences for survival in the mid-Columbia River
Source: PLoS One. 2021 May 10;16(5):e0250831. doi: 10.1371/journal.pone.0250831 (PMC8109777; doi:10.1371/journal.pone.0250831)
Supplement: S4 Table — AICc model selection table for the probability of survival (pSurv). Variables considered included ocean age (A), reartype origin (O), juvenile transportation history (J), a linear effect for annual harvest (H), a smoother for the predicted probability of delay s(pDelay^), and a random effect for year y. Coefficient values given for intercept and linear effect of H. Number of parameters represented by np. (DOCX) [file pone.0250831.s004.docx]

**S4 Table: Model selection for survival models.** AICc model selection table for the probability of survival (*pSurv*). Variables considered included ocean age (*A*), reartype origin (*O*), juvenile transportation history (*J*), a linear effect for annual harvest (*H*), a smoother for the predicted probability of delay s($\hat{pDelay}$), and a random effect for year *y*. Coefficient values given for intercept and linear effect of *H*. Number of parameters represented by *np*.

| **Int.** | ***A*** | ***O*** | ***J*** | ***H*** | ***s(***$\hat{\boldsymbol{pDelay}}$***)*** | ***y*** | **np** | **delta** |
| --- | --- | --- | --- | --- | --- | --- | --- | --- |
| *Middle Columbia* | | | | | | | | |
| 1.43 | + | + | NA |  | + | + | 10 | 0 |
| 1.48 | + | + | NA | -0.33 | + | + | 11 | 1.02 |
| 1.60 | + |  | NA |  | + | + | 9 | 3.76 |
| 1.48 | + |  | NA | 0.75 | + | + | 10 | 4.83 |
| *Snake Early A-index* | | |  |  |  |  |  |  |
| 1.29 | + | + | + |  | + | + | 11 | 0 |
| 1.53 | + | + | + | -1.41 | + | + | 12 | 0.39 |
| 1.33 | + |  | + |  | + | + | 11 | 4.27 |
| 1.59 | + |  | + | -1.56 | + | + | 12 | 4.49 |
| *Upper Columbia* | |  |  |  |  |  |  |  |
| 1.18 | + | + | NA |  | + | + | 12 | 0 |
| 1.03 | + | + | NA | 0.95 | + | + | 13 | 0.59 |
| 1.19 | + |  | NA |  | + | + | 12 | 0.82 |
| 1.00 | + |  | NA | 1.19 | + | + | 12 | 1.33 |
| *Sal/Clear A-index* | |  |  |  |  |  |  |  |
| 3.14 | + |  | + | -7.71 | + | + | 8 | 0 |
| 1.81 | + |  | + |  | + | + | 10 | 0.6 |
| 3.17 | + | + | + | -7.83 | + | + | 9 | 1.89 |
| 1.81 | + | + | + |  | + | + | 11 | 2.56 |
| 3.18 | + |  | + | -8.08 | + |  | 5 | 2.81 |
| 3.21 | + | + | + | -8.19 | + |  | 6 | 4.62 |
| *Sal/Clear B-index* | |  |  |  |  |  |  |  |
| 2.25 |  | + |  | -5.05 | + | + | 10 | 0 |
| 2.31 | + | + |  | -4.93 | + | + | 11 | 1.35 |
| 1.39 |  | + |  |  | + | + | 10 | 1.46 |
| 2.28 |  | + | + | -5.13 | + | + | 11 | 1.75 |
| 1.47 | + | + |  |  | + | + | 11 | 2.81 |
| 2.33 | + | + | + | -5.01 | + | + | 12 | 3.07 |
| 1.40 |  | + | + |  | + | + | 11 | 3.26 |
| 2.43 |  |  |  | -5.73 | + | + | 9 | 3.55 |
| 1.48 | + | + | + |  | + | + | 12 | 4.59 |
| 2.49 | + |  |  | -5.57 | + | + | 10 | 4.64 |
