# Supplementary figures and images for: Environmentally triggered shifts in steelhead migration behavior and consequences for survival in the mid-Columbia River
Source: PLoS One. 2021 May 10;16(5):e0250831. doi: 10.1371/journal.pone.0250831 (PMC8109777; doi:10.1371/journal.pone.0250831)

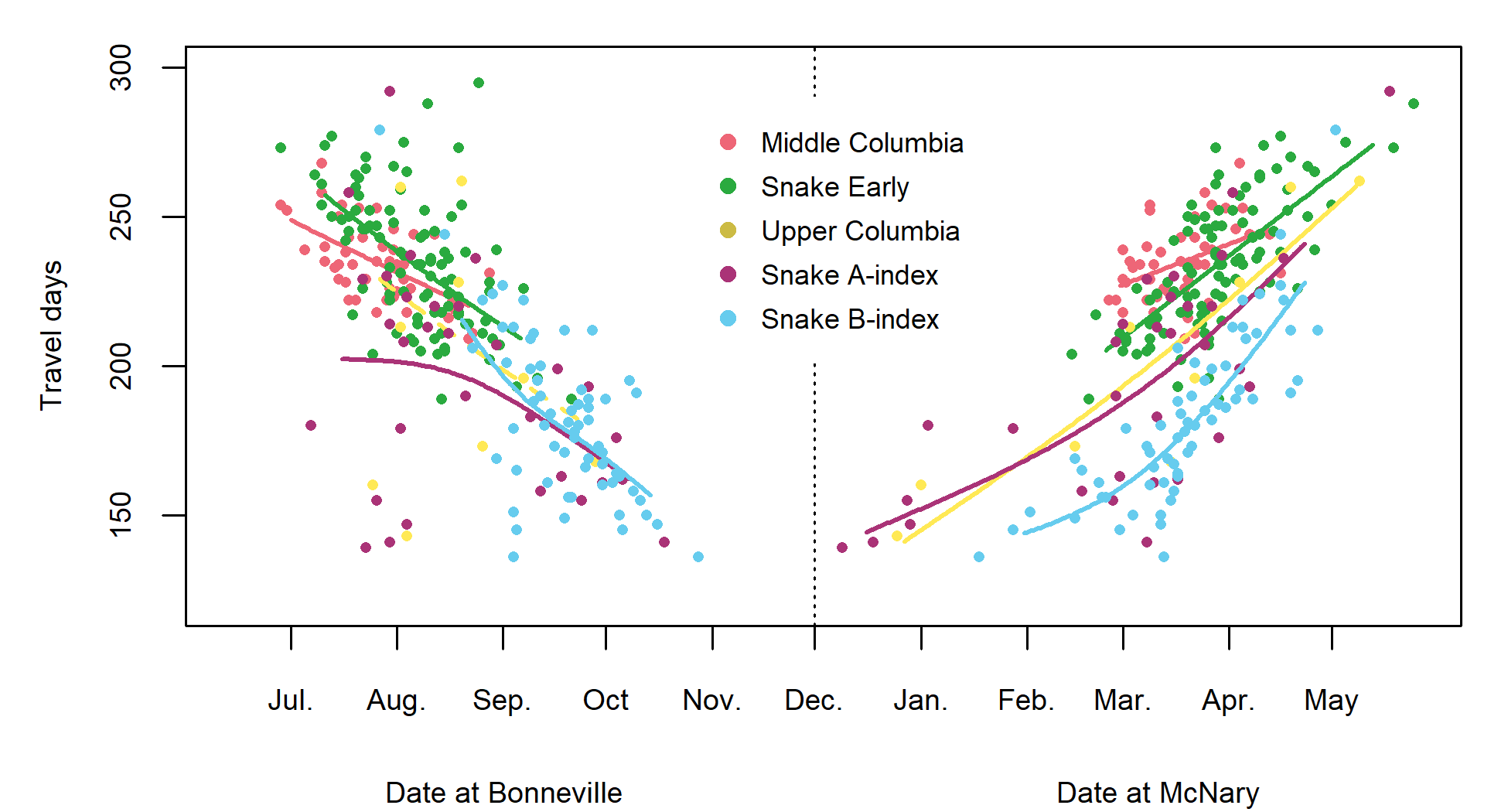

Supplement: S1 Fig — Travel times and arrival dates at Bonneville Dam and McNary Dam for overwintering fish. Smoothed relationships between arrival dates and travel times at each dam are shown. (TIFF) [file pone.0250831.s005.tiff]

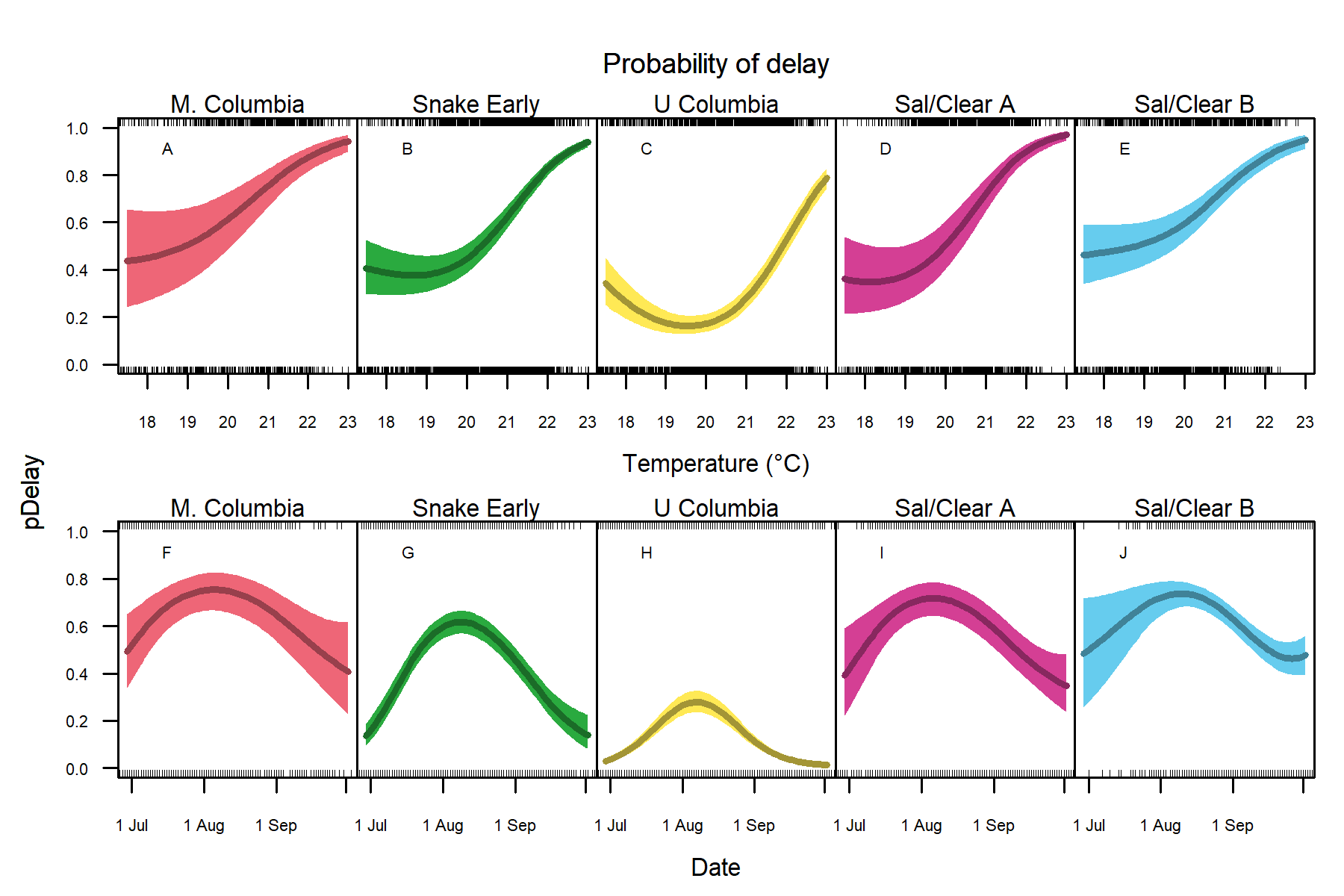

Supplement: S2 Fig — Fitted effects for selected continuous variables (Temperature and Date) from probability of survival models showing 95% confidence intervals. (TIFF) [file pone.0250831.s006.tiff]

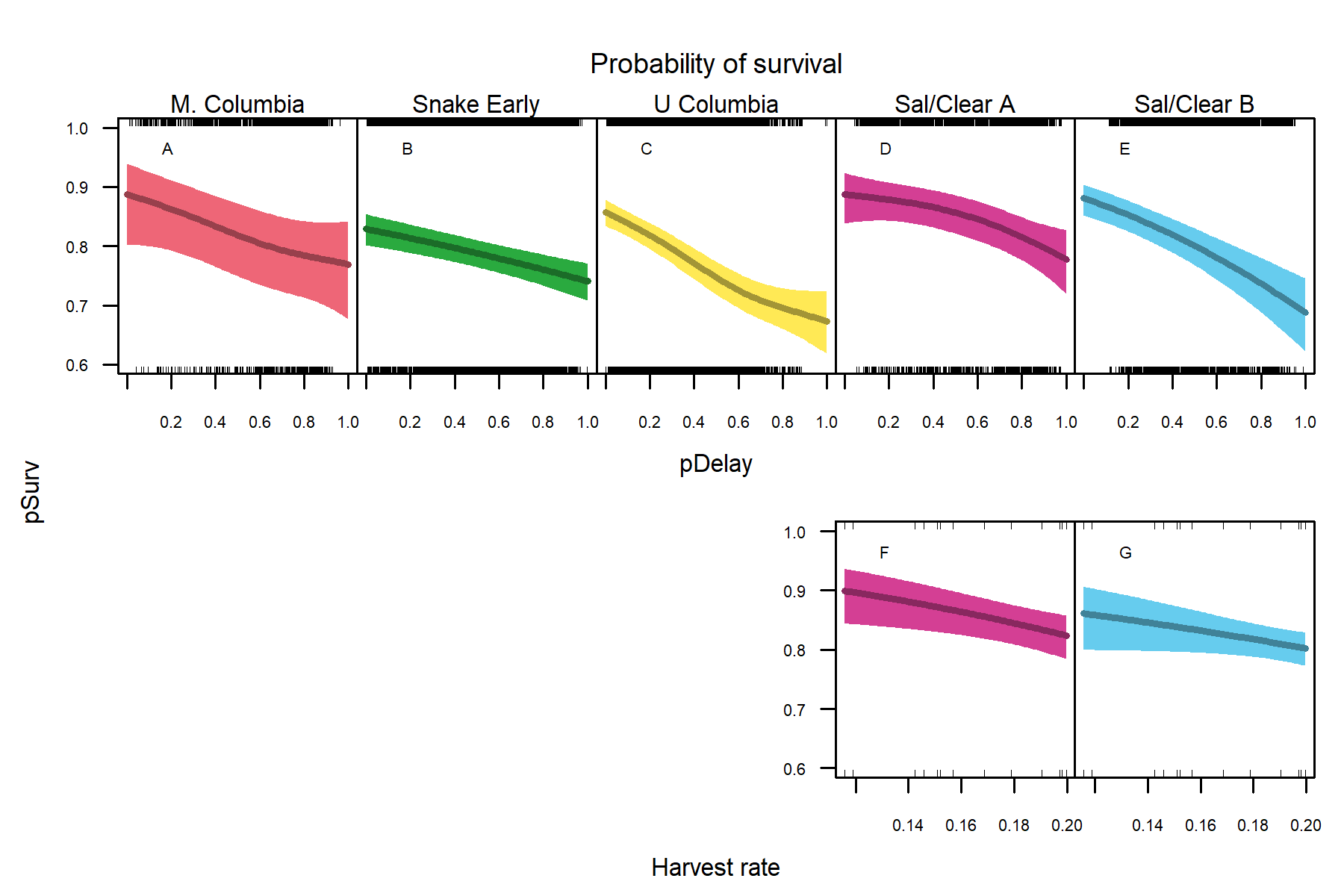

Supplement: S3 Fig — Fitted effects for selected continuous variables (pDelay and Harvest Rate) from probability of survival models showing 95% confidence intervals. (TIFF) [file pone.0250831.s007.tiff]
